# Supplementary material for: Optoelectronic system and device integration for quantum-dot light-emitting diode white lighting with computational design framework
Source: Nat Commun. 2022 Aug 3;13:4189. doi: 10.1038/s41467-022-31853-9 (PMC9349286; doi:10.1038/s41467-022-31853-9)
Supplement: Supplementary file 1 — Supplementary Information [file 41467_2022_31853_MOESM1_ESM.pdf]

Supplementary Information

Optoelectronic System and Device Integration for Quantum-Dot Light-Emitting Diode White Lighting with Computational Design Framework

Chatura Samarakoon[1], Hyung Woo Choi[1], Sanghyo Lee[1], Xiang-Bing Fan[1], Dong-Wook Shin[1], Sang Yun Bang[1], Jeong-Wan Jo[1], Limeng Ni[1], Jiajie Yang[1], Yoonwoo Kim[1], Sung-Min Jung[1*], Luigi G. Occhipinti[1], Gehan A. J. Amaratunga[1], and Jong Min Kim[1]

[1]*Electrical Engineering Division, Department of Engineering, University of Cambridge, 9 JJ Thomson Ave, Cambridge, CB3 0FA, United Kingdom*

* Corresponding author: Sung-Min Jung

E-mail address: sj569@cam.ac.uk

I. Supplementary Figures

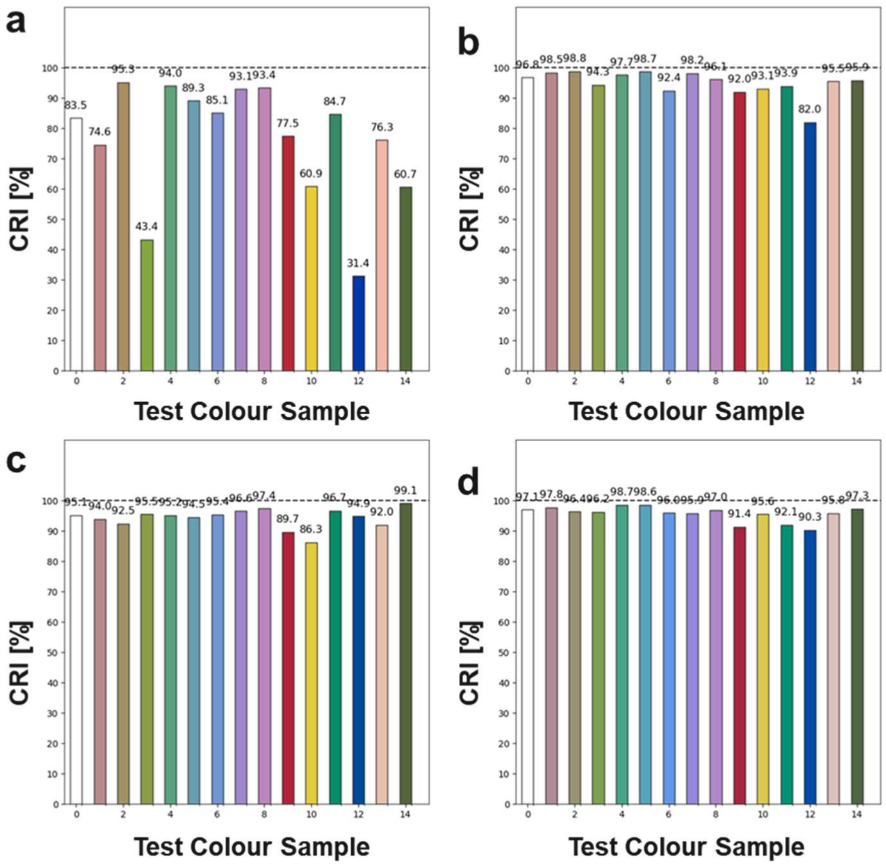

Supplementary Fig. 1: Colour rendering index (CRI) bar charts for the white lighting optimised by the colour optimisation process. a 3-, **b** 4-, **c** 5-, and **d** 6-primary coloured lighting.

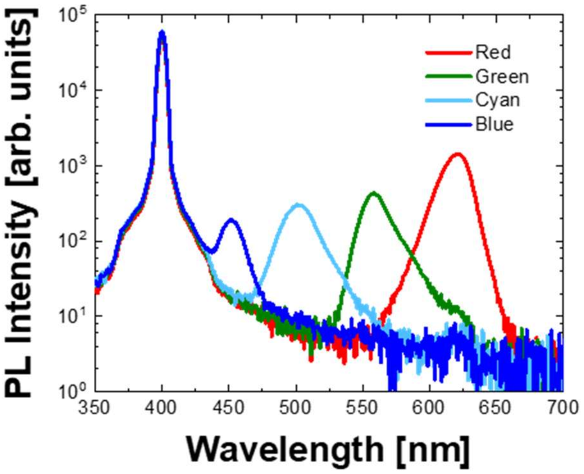

Supplementary Fig. 2: Photoluminescence (PL) spectral intensities of red, green, cyan, and blue QDs used for the fabrication of the QD-LED based white lighting system. PL peak wavelengths of the red, green, cyan, and blue QDs are measured to be 621 nm, 558 nm, 502 nm, and 452 nm, respectively.

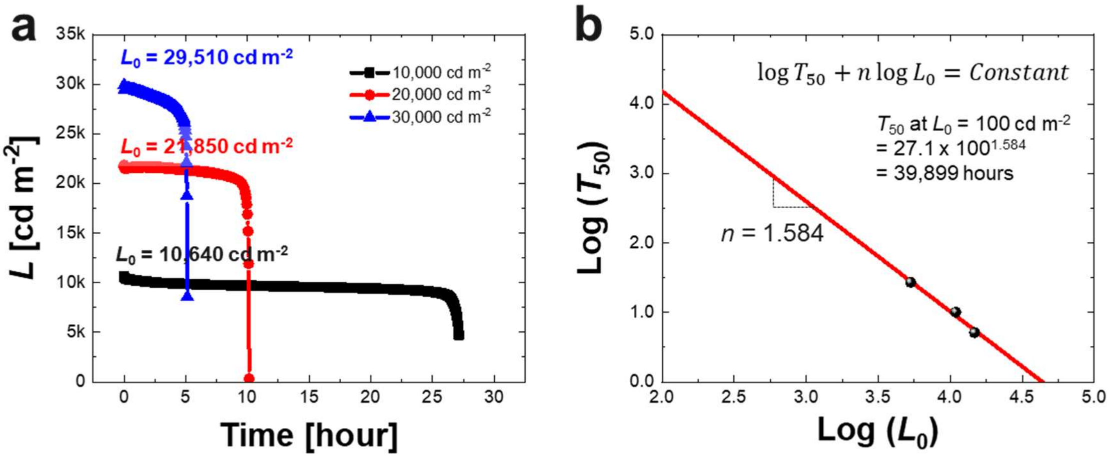

Supplementary Fig. 3: Measurement of device lifetime for the QD-LED fabricated by the transfer printing process. a Time-dependent luminance variation of the red QD-LED device at various initial luminances (L_0). **b** Linear extrapolation of Log(T_{50}) for Log(L_0), where T_{50} is the time to reach 50% of an initial luminance L_0.

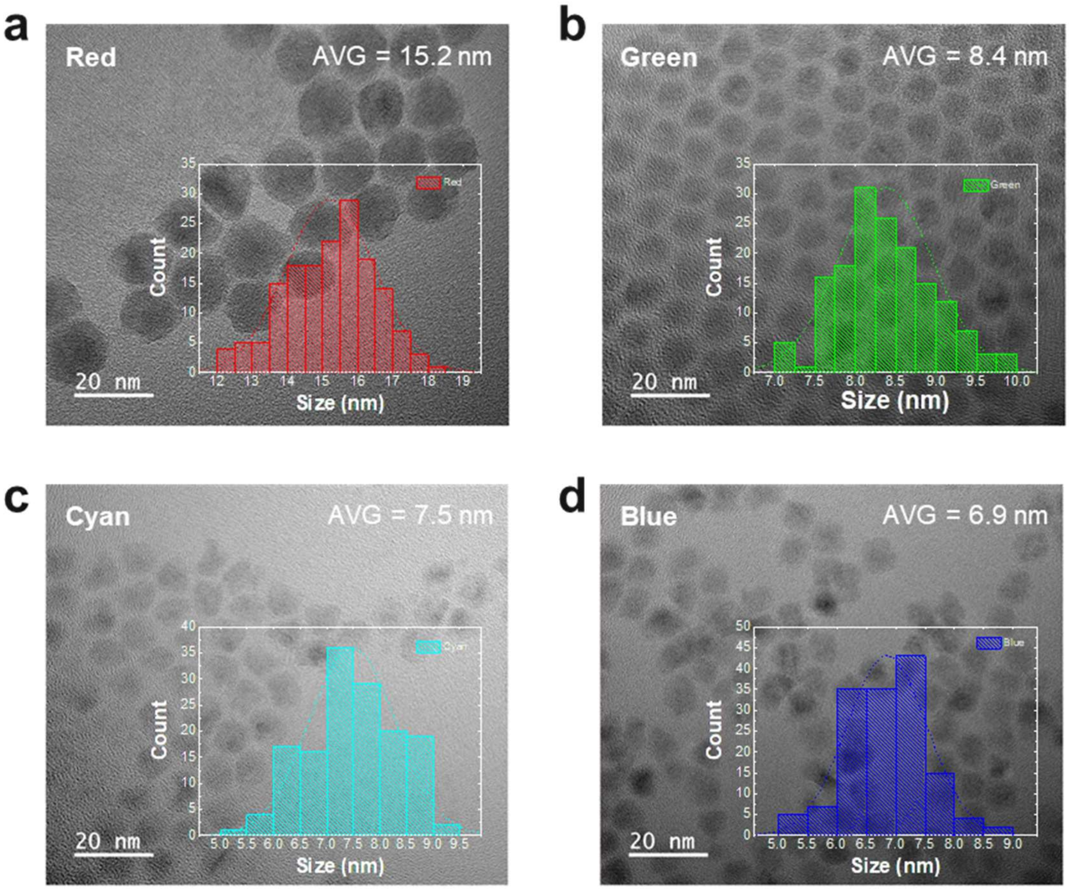

Supplementary Fig. 4: TEM images and the particle size distribution of QDs used in the fabrication of QD-LEDs. a Red, **b** green, **c** cyan, and **d** blue QDs. The measured particle sizes are used for the charge transport simulation of the monochromatic QD-LED devices.

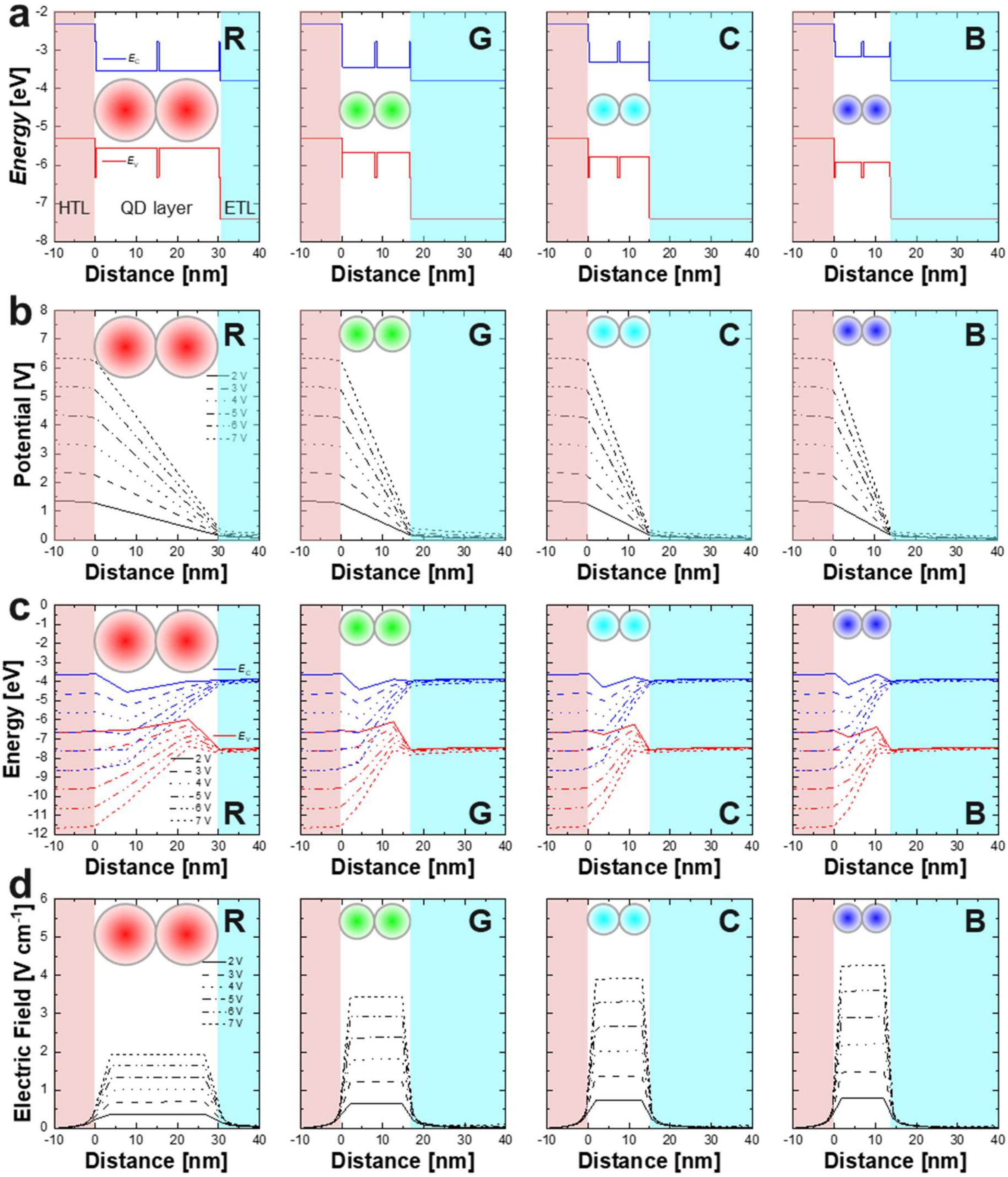

Supplementary Fig. 5: Simulated field distribution around the quantum dot (QD) layer for red (R), green (G), cyan (C), and blue (B) QD-LED devices. a Flat-band energy diagram for the distance from the interface between the hole transport layer (HTL) and the QD layer. **b** Potential distribution, **c** conduction and valence band (E_C and E_V) energy distributions, and **d** electric field distribution obtained from the charge transport simulation under the various applied voltages.

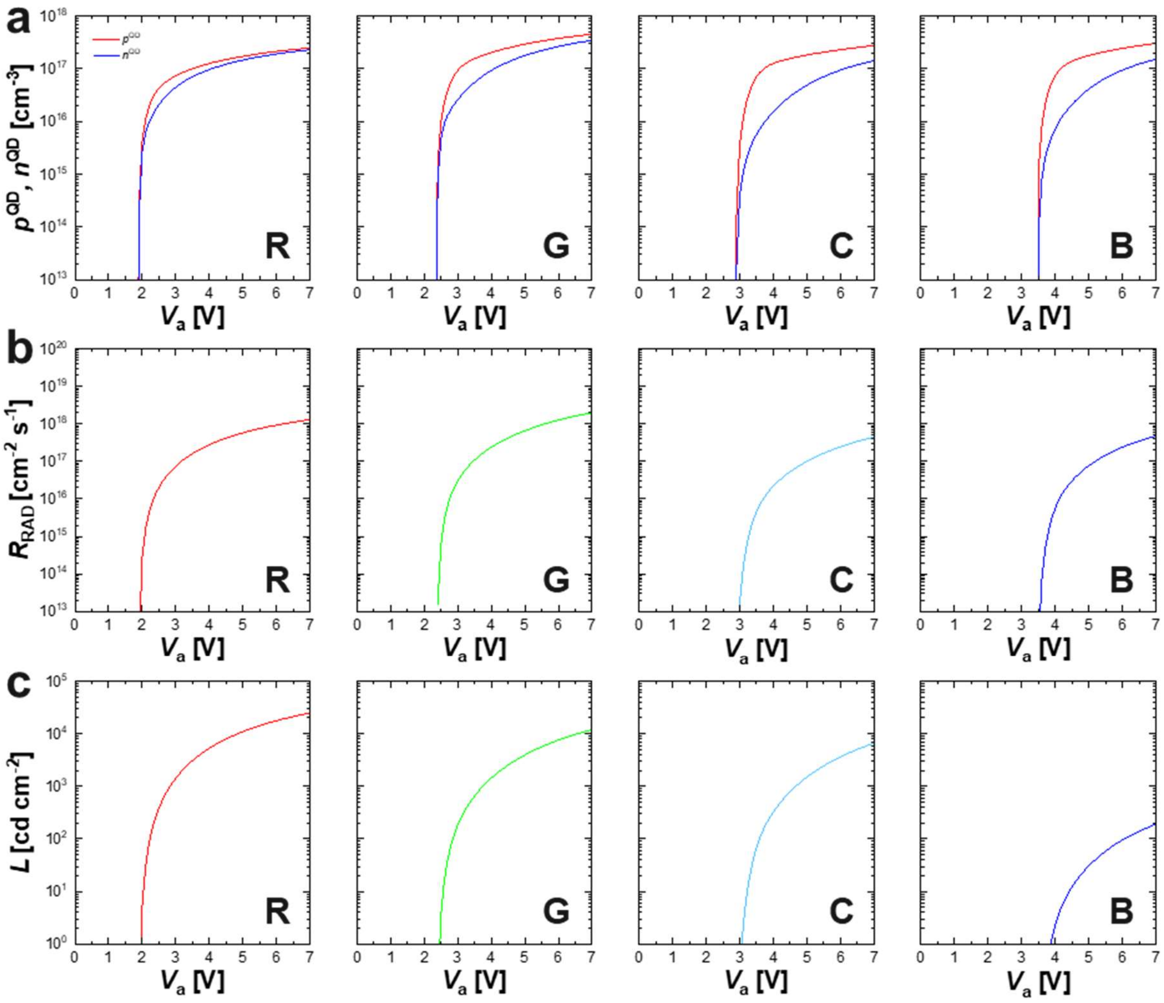

Supplementary Fig. 6: Simulated light emission behaviours of the monochromatic red (R), green (G), cyan (C), and blue (B) QD-LED devices for the applied voltage (V_a). a Hole and electron densities (p^{QD} and n^{QD}) at QD layer. **b** Radiative recombination rate per unit area (R_{RAD}). **c** Luminance (L) of the monochromatic QD-LED devices calculated from the simulated radiative recombination rate per unit area, R_{RAD}.

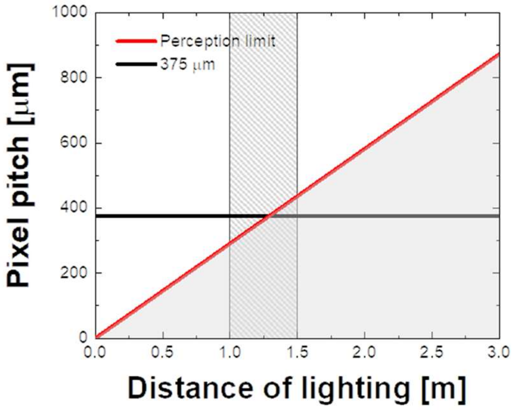

Supplementary Fig. 7: Perception limit of pixel group pitch for the distance of lighting from the user. The red line shows the perception limitation of pixel pitch for the distance of lighting. The pixel having the pitch below the perception limitation cannot be recognized by the user at the given distance.

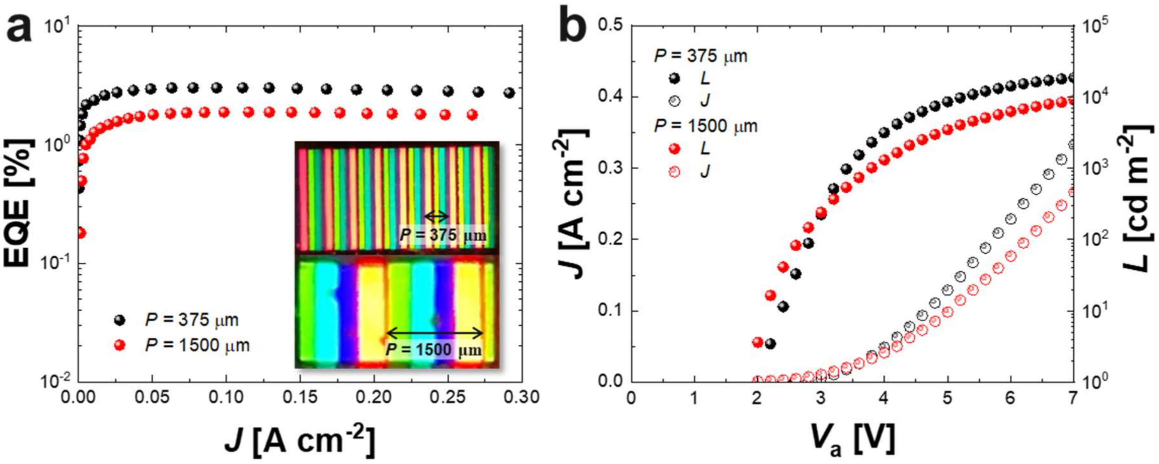

Supplementary Fig. 8: Experimental electro-optical properties of the reference lighting systems having identical subpixel pattern widths for the horizontal pitches *P* of 375 µm and 1500 µm. a External quantum efficiency (EQE)-current density (*J*) curves of the reference lighting systems for the pixel pitch *P* = 375 µm and 1500 µm. Insets are the snapshots of the respective lighting systems in electroluminescence (EL) operation. **b** Current density – voltage (V_a) – luminance (*L*) curves of the lighting system having identical subpixel pattern widths for the pitches of 375 µm and 1500 µm.

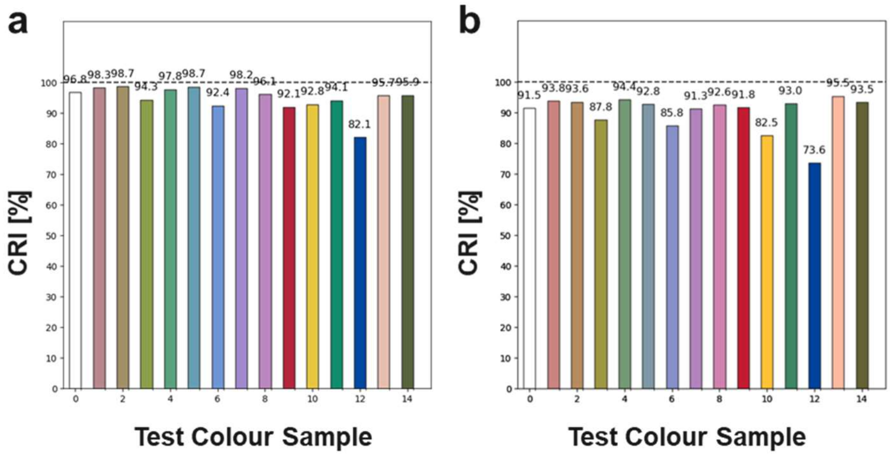

Supplementary Fig. 9: Colour rendering index (CRI) Bar charts of the optimised 4-primary white lighting systems. a Simulated CRI values for 14 test colour samples. The white bar in the chart is an average of CRIs for the test colour samples from 1 to 8. **b** Experimental CRI values obtained from the fabricated device.

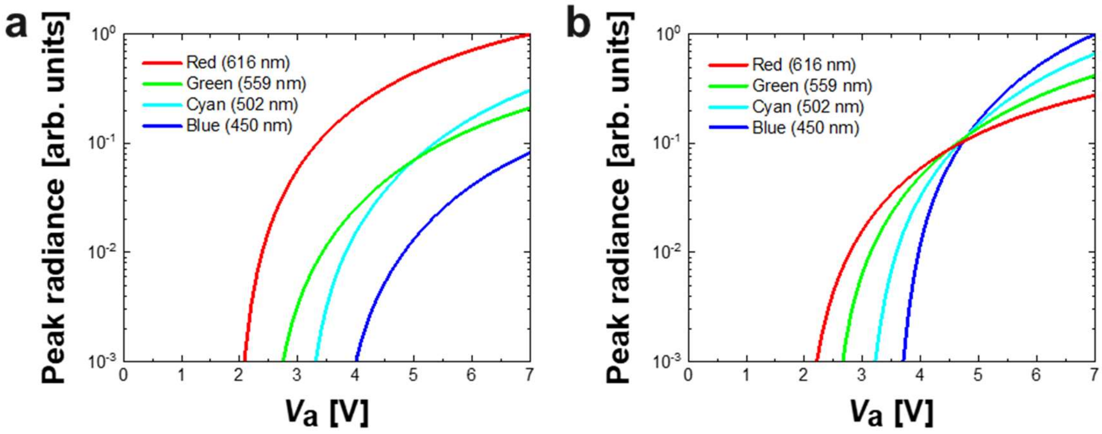

Supplementary Fig. 10: Emission contribution for the applied voltage (V_a) at peak wavelengths of each QD pattern. Variation of simulated peak radiance at peak wavelengths of 616 nm (red), 559 nm (green), 502 nm (cyan), and 450 nm (blue) QD patterns for the lighting systems having **a** identical emission widths and **b** optimised emission widths.

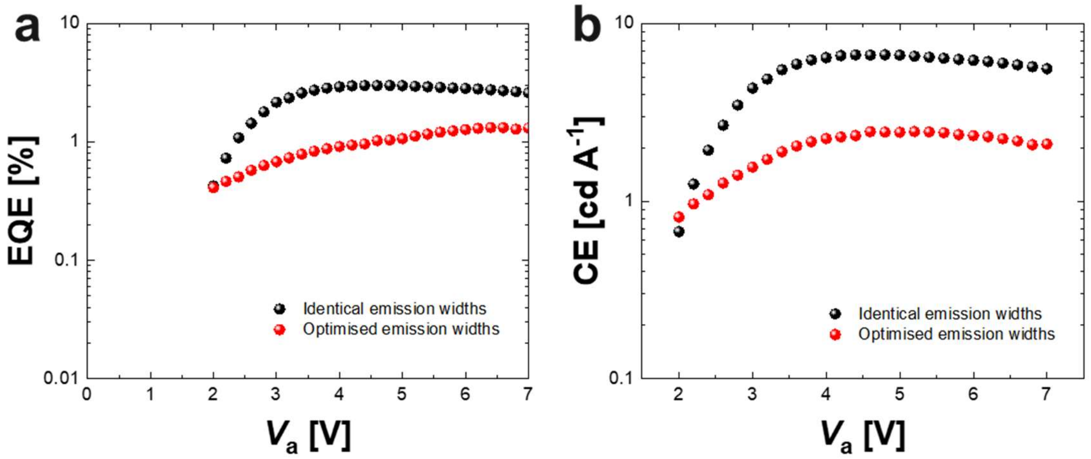

Supplementary Fig. 11: Experimental EL performances of the white QD-LED lighting systems having the optimised (red) and identical (black) emission widths. a External quantum efficiency (EQE) and **b** current efficiency (CE) of the white lighting systems for the applied voltage (V_a).

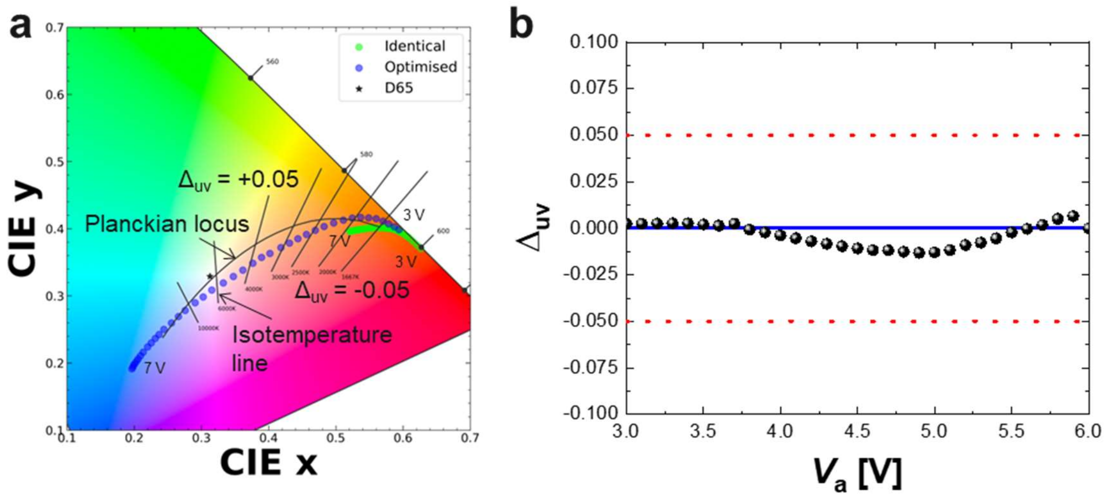

Supplementary Fig. 12: Experimental colour variation of the optimised QD-LED white lighting system. a Colour locus for the applied voltages from 3 V to 7 V with 0.1 V step. **b** Distance from Planckian locus (Δ_{uv}) for the applied voltage (V_a).

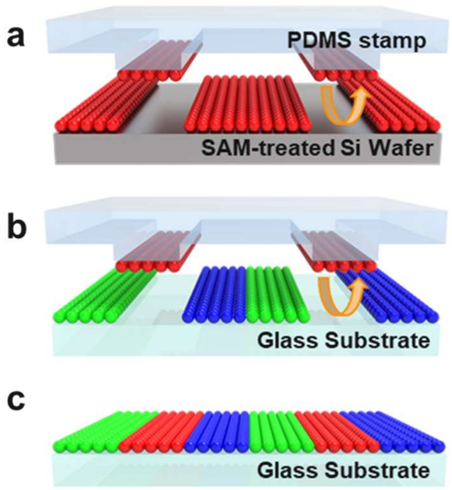

Supplementary Fig. 13: Schematic illustration of a transfer printing technique. a Pick-up process of QD layer from self-assembled monolayer (SAM) treated silicon (Si) wafer by using polydimethylsiloxane (PDMS) stamp. **b** Transfer printing process to the pre-processed glass substrate. **c** Final QD patterns.

II. Supplementary Tables

Supplementary Table 1: Material parameters for the transport layers and QD shell used in the simulation.

Parameters	HIL	HTL	ETL	QD Shell
Materials	PEDOT:PSS	TFB	MZO	ZnS
Thickness [nm]	20	20	40	0.25
E_{C0}[1-3] [eV]	-3.60	-2.30	-3.80	-2.78
E_{V0}[1-3] [eV]	-5.17	-5.30	-7.40	-6.32
μ_p[4,5] [cm^2 V^{-1} s^{-1}]	3.2×10^{-4}	$2.0e\times10^{-3}$		-
μ_n[6] [cm^2 V^{-1} s^{-1}]			2.0×10^{-3}	-
ε_r[2,4,7]	3.0	3.5	8.5	-
N_d[8] [cm^{-3}]	-	-	1.0×10^{17}	-
N_a[8,9] [cm^{-3}]	2.81×10^{19}	1.0×10^{17}	-	-
τ[1,8] [μs]	1.0	1.0	1.0	-

Supplementary Table 2: Performance summary of previous studies on QD-LED based white lighting systems. R, G, C, B, and W denotes the red, green, cyan, blue, and white, respectively.

References	Device features	Luminance [cd/m^2]	EQE [%]	Current Efficiency [cd/A]	CIE$_{xy}$ (@ white)	CRI [%]	CCT [K]
	CIE D65 standard illuminant	-	-	-	(0.31, 0.33)	100	6500
This work	Transfer printed white QD-LEDs with RGB pixelation	19,670 (R)<br>10,370 (G)<br>8,070 (C)<br>442 (B)<br>3,285 (W)	4.3 (R)<br>0.6 (G)<br>1.0 (C)<br>0.3 (B)<br>1.4 (W)	2.5 (W)	(0.33, 0.32)	92	1612 – 8903
Choi *et al.*[10]	Transfer printed white QD-LEDs with RGB patterning	4000 (W)	1.6 (W)	-	(0.39, 0.38)	-	-
Kim *et al.*[11]	Transfer printed white QD-LEDs with RGB stacking	3380 (W)	-	0.4 (W)	(0.36, 0.37)	-	-
Bae *et al.*[12]	Spin coated white QD-LEDs with RGB mixing	3220 (W)	0.9 (W)	-	(0.31, 0.32)	92	6874
Kim *et al.*[13]	Transfer printed monochromatic QD-LEDs with RGB patterning	16,380 (R)<br>6,425 (G)<br>423 (B)	-	4 (R)<br>0.5 (G)<br>0.04 (B)	-	-	-
Nam *et al.*[14]	Transfer printed monochromatic QD-LEDs with thermodynamic route	14,063 (G)	3.3 (G)	14.8 (G)	-	-	-
Kim *et al.*[15]	Transfer printed monochromatic QD-LEDs with multi-layer pick-up	1,200 (R)<br>600 (G)<br>30 (B)	2.3 (R)<br>1.4 (G)<br>0.03 (B)	-	-	-	-

Supplementary Table 3: Material parameters for red, green, cyan, and blue QDs used in the simulation.

Parameters	QDs			
	Red (CdSe/ZnS)	Green (CdSe/ZnS)	Cyan (CdSe/ZnS)	Blue (CdSe/ZnS)
ε_r[16]	9.4	9.4	9.4	9.4
Peak Wavelength [nm]	616	559	502	450
FWHM	20	20	20	20
Diameter [nm]	15.2	8.4	7.5	6.9
Number of QD layers	2	2	2	2
LUMO [eV]	-3.56	-3.45	-3.33	-3.19
HOMO [eV]	-5.54	-5.65	-5.77	-5.91
Optical Bandgap [eV]	1.98	2.20	2.44	2.72
μ_p^{QD} [cm^2 V^{-1} s^{-1}]	2.6×10^{-5}	2.6×10^{-5}	2.6×10^{-5}	2.6×10^{-5}
μ_n^{QD} [cm^2 V^{-1} s^{-1}]	2.6×10^{-5}	2.6×10^{-5}	2.6×10^{-5}	2.6×10^{-5}
σ_p	0.13	0.018	0.0085	0.009
σ_n	0.13	0.018	0.0085	0.009
η_{QD}	0.32	0.04	0.23	0.05
γ [cm^3 s^{-1}]	1.0×10^{-11}	1.0×10^{-11}	1.0×10^{-11}	1.0×10^{-11}
τ [μs]	2.24	1.20	0.136	0.133
C [cm^6 s^{-1}]	1.15×10^{-29}	0.34×10^{-29}	4.3×10^{-29}	3.16×10^{-29}
Q	4.41	5.94	0.56	0.65

Supplementary Table 4: Summary of QD-LED device lifetimes reported from recent publications.

References	QD film process	QD Colour	Lifetime [hour]	Condition
Yang *et al.*[17]	Spin coating	Red<br>Green<br>Blue	90,000<br>300,000<br>1,000	T_{50} at 100 cd m^{-2}<br>T_{50} at 100 cd m^{-2}<br>T_{50} at 100 cd m^{-2}
Dai *et al.*[18]	Spin coating	Red	100,000	T_{50} at 100 cd m^{-2}
Pu *et al.*[19]	Spin coating	Red<br>Blue	90,000<br>10,000	T_{50} at 100 cd m^{-2}<br>T_{50} at 100 cd m^{-2}
Weiran *et al.*[20]	Spin coating	Red	2,200,000	T_{50} at 100 cd m^{-2}
Chen *et al.*[21]	Spin coating	Red<br>Blue	800<br>23	T_{90} at 1000 cd m^{-2}<br>T_{50} at 1000 cd m^{-2}
Choi *et al.*[10]	Transfer printing	Green	41.7<br>> 1900	T_{50} at 4554 cd m^{-2}<br>T_{50} at 100 cd m^{-2}
Kim *et al.*[13]	Transfer printing	Red	76.5<br>> 800	T_{50} at 1050 cd m^{-2}<br>T_{50} at 100 cd m^{-2}
Kim *et al.*[15]	Transfer printing	Red	35<br>> 700	T_{50} at 2037 cd m^{-2}<br>T_{50} at 100 cd m^{-2}
This work	Transfer printing	Red	39,899	T_{50} at 100 cd m^{-2}

Supplementary References

1. Vahabzad, F., Rostami, A., Dolatyari, M., Rostami, G. & Amiri, I. S. Solution-processed QD-LEDs in visible range: Modulation bandwidth enhancement. *Physica B* **574**, 411667 (2019).
2. Han, Y. J., An, K., Kang, K. T., Ju, B.-K. & Cho, K. H. Optical and electrical analysis of annealing temperature of high-molecular weight hole transport layer for quantum-dot light-emitting diodes. *Sci. Rep.* **9**, 10385 (2019).
3. Li, D. *et al.* Enhanced efficiency of InP-based red quantum dot light-emitting diodes. *ACS Appl. Mater. Interfaces* **11**, 34067–34075 (2019).

4]. Xu, B. *et al.* Functional solid additive modified PEDOT:PSS as an anode buffer layer for enhanced photovoltaic performance and stability in polymer solar cells. *Sci. Rep.* **7**, 45079 (2017).

5. Zhao, Y. *et al.* Composite hole transport layer consisting of high-mobility polymer and small molecule with deep-lying HOMO level for efficient quantum dot light-emitting diodes. *IEEE Electron Device Lett.* **41**, 80–83 (2020).
6. Wang, F. *et al.* Achieving balanced charge injection of blue quantum dot light-emitting diodes through transport layer doping strategies. *J. Phys. Chem. Lett.* **10**, 960–965 (2019).
7. Vallisree, S., Thangavel, R. & Lenka, T. R. Modelling, simulation, optimization of Si/ZnO and Si/ZnMgO heterojunction solar cells. *Mater. Res. Express* **6**, 025910 (2018).
8. Kumar, B., Campbell, S. A. & Paul Ruden, P. Modeling charge transport in quantum dot light emitting devices with NiO and ZnO transport layers and Si quantum dots. *J. Appl. Phys.* **114**, 044507 (2013).
9. Yan, F., Parrott, E. P. J., Ung, B. S.-Y. & Pickwell-MacPherson, E. Solvent doping of PEDOT/PSS: Effect on terahertz optoelectronic properties and utilization in terahertz devices. *J. Phys. Chem. C* **119**, 6813–6818 (2015).

10. Choi, M. K. *et al.* Wearable red–green–blue quantum dot light-emitting diode array using high-resolution intaglio transfer printing. *Nat. Commun.* **6**, 7149 (2015).

11. Kim, T.-H. *et al.* Heterogeneous stacking of nanodot monolayers by dry pick-and-place transfer and its applications in quantum dot light-emitting diodes. *Nat. Commun.* **4**, 2637 (2013).

12. Bae, W. K. *et al.* R/G/B/Natural white light thin colloidal quantum dot-based light-emitting devices. *Adv. Mater.* **26**, 6387–6393 (2014).

13. Kim, T.-H. *et al.* Full-colour quantum dot displays fabricated by transfer printing. *Nat. Photonics* **5**, 176–182 (2011).

14. Nam, T. W. *et al.* Thermodynamic-driven polychromatic quantum dot patterning for light-emitting diodes beyond eye-limiting resolution. *Nat. Commun.* **11**, 3040 (2020).

15. Kim, B. H. *et al.* Multilayer transfer printing for pixelated, multicolor quantum dot light-emitting diodes. *ACS Nano* **10**, 4920–4925 (2016).

16. Canali, C., Nava, F., Ottaviani, G. & Paorici, C. Hole and electron drift velocity in CdSe at room temperature. *Solid State Communications* **11**, 105–107 (1972).

17. Yang, Y. *et al.* High-efficiency light-emitting devices based on quantum dots with tailored nanostructures. *Nat. Photonics* **9**, 259–266 (2015).

18. Dai, X. *et al.* Solution-processed, high-performance light-emitting diodes based on quantum dots. *Nature* **515**, 96–99 (2014).

19. Pu, C. *et al.* Electrochemically-stable ligands bridge the photoluminescence-electroluminescence gap of quantum dots. *Nat. Commun.* **11**, 937 (2020).

20. Cao, W. *et al.* Highly stable QLEDs with improved hole injection via quantum dot structure tailoring. *Nat. Commun.* **9**, 2608 (2018).

21. Chen, S. *et al.* On the degradation mechanisms of quantum-dot light-emitting diodes. *Nat. Commun.* **10**, 765 (2019).
